# Supplementary material for: Thinking about life in COVID-19: An exploratory study on the influence of temporal framing on streams-of-consciousness
Source: PLoS One. 2023 Apr 28;18(4):e0285200. doi: 10.1371/journal.pone.0285200 (PMC10146429; doi:10.1371/journal.pone.0285200)
Supplement: S1 Table — The table below features the seven selected components, the ten most influential LIWC features (ordered from most to least influential, based on their absolute values), and their raw loading scores. Positive loading scores best characterize the during-pandemic condition, while negative loading scores best characterize the pre-pandemic condition. (DOCX) [file pone.0285200.s001.docx]

| **PC1 (*)** | | **PC4 (*)** | |
| --- | --- | --- | --- |
| **LIWC category** | **Loading score** | **LIWC category** | **Loading score** |
| Health | 0.241 | Fulfill | -0.231 |
| Illness | 0.234 | Prosocial | -0.204 |
| Analytic | 0.223 | Exclamation | -0.202 |
| Pronoun | -0.212 | I | -0.183 |
| Neg. tone | 0.204 | Insight | -0.18 |
| Article | 0.204 | Space | 0.172 |
| Social refs | -0.186 | Aux. verb | 0.171 |
| Social | -0.182 | Social behavior | -0.17 |
| Personal Pronoun | -0.177 | Perception | 0.166 |
| Physical | 0.171 | Allure | 0.162 |
| **PC5 (**)** | | **PC10 (*)** | |
| **LIWC category** | **Loading score** | **LIWC category** | **Loading score** |
| Pos. tone | 0.267 | Comm | -0.244 |
| Want | 0.254 | Impers. pronoun | -0.2215 |
| Tone | 0.25 | Memory | -0.21 |
| Discrepancy | 0.244 | Conjunctions | 0.203 |
| Pos. emotion | 0.236 | Acquire | 0.197 |
| Emotion | 0.192 | Swear | 0.158 |
| Past focus | -0.184 | Risk | 0.154 |
| Authentic | -0.181 | Sad emotion | -0.154 |
| Lack | 0.179 | Leisure | -0.153 |
| Personal pronoun | -0.173 | Lifestyle | -0.15 |
| **PC11 (*)** | | **PC13 (*)** | |
| **LIWC category** | **Loading score** | **LIWC category** | **Loading score** |
| You | 0.243 | Cause | 0.235 |
| Achieve | -0.229 | Mental | -0.228 |
| Death | -0.206 | Visual | -0.197 |
| Work | -0.191 | Time | 0.196 |
| Male | 0.177 | Perception | -0.19 |
| Cognition | -0.174 | Past focus | -0.18 |
| Lifestyle | -0.17 | Future focus | 0.171 |
| Q mark | -0.166 | Conjunctions | 0.165 |
| Space | 0.162 | She/he | 0.16 |
| Certitude | -0.161 | Need | 0.158 |
| **PC18** | |  |  |
| **LIWC category** | **Loading score** |  |  |
| Moral | 0.318 |  |  |
| Impers. Pronoun | 0.295 |  |  |
| Need | -0.265 |  |  |
| You | -0.208 |  |  |
| Personal pronoun | -0.185 |  |  |
| Certitude | 0.169 |  |  |
| Motion | 0.169 |  |  |
| Words per sentence | 0.167 |  |  |
| Apostrophes | -0.159 |  |  |
| Affect | 0.159 |  |  |
