# Supplementary material for: Thinking about life in COVID-19: An exploratory study on the influence of temporal framing on streams-of-consciousness
Source: PLoS One. 2023 Apr 28;18(4):e0285200. doi: 10.1371/journal.pone.0285200 (PMC10146429; doi:10.1371/journal.pone.0285200)
Supplement: Attachment — Submitted filename: Response to Reviewers.docx [file pone.0285200.s003.docx]

Dear Dr. Ptaszynski,

Thank you for inviting us to complete a revision and resubmission of our manuscript, entitled “Thinking about life in COVID-19: An exploratory study on the influence of temporal framing on streams of consciousness.” In our latest review, we received a comment about the exploratory nature of the study. We hope to clarify this point, as we believe this was addressed in our first revision, and want to make sure we are not misunderstanding the reviewer’s request.

We believe through the revision process we have been able to improve the manuscript greatly, and are grateful to you and the reviewers for your time and input.

Sincerely,

Constance Bainbridge and Rick Dale

Reviewer #1:

The study seems quantitative, but it was dealt with qualitatively, and therefore the results are more like suggestions and not facts, which confirms that if the application is repeated again on these students, will the same results be obtained? I expect not.

Therefore, I suggest adding the word exploratory study in the title of the study

**Response**: We hope to clarify this point: we had updated the title of the manuscript to include “exploratory study” in our previous revision, honoring this very helpful comment from Reviewer #1. We noticed the “short title” we submitted into the system still used the former title, so we have additionally updated the short title to now read “Thinking about life in COVID-19: An exploratory study on the influence of temporal framing.” If there is an alternate spot in the manuscript this comment intended to address, we are happy to make any additional updates to make sure this aspect of the research is clear. We have resubmitted again and double checked that “exploratory” is indeed in the title of the manuscript. If this is not visible to reviewers or editor, we welcome any advice about correcting it. Thank you again for your suggestion!

Reviewer #2: Dear authors, I'm very impressed by your work, congratulations! Thank you for considering my suggestions and for your kind remarks on them. I hope to see your paper published soon.

All the best!

Reviewer #3: The authors have addressed all of my comments in the previous round, and I believe the manuscript, which was already in good shape, is even stronger now. In particular, I found the additional paragraphs on interpreting the PCs and network analysis were very helpful. Overall, I think this will make a very nice contribution to PLOS ONE, and I appreciate the opportunity to review this work!

**Response to all reviews**: Thank you once again for your valuable feedback in strengthening the manuscript!
